# Depression in elderly patients hospitalized in psychiatry

**DOI:** 10.1192/j.eurpsy.2021.893

**Published:** 2021-08-13

**Authors:** N. Halouani, R. Ouali, E. Jemal, M. Turki, S. Ellouze, R. Charf, L. Aribi, J. Aloulou

**Affiliations:** 1 Psychiatry B Chu Hedi Chaker, Tunisia, psychiatry B, sfax, Tunisia; 2 Psychiatry (b), Hedi Chaker University hospital, sfax, Tunisia; 3 Psychiatry (b), Hedi Chaker University hospital, Sfax, Tunisia; 4 Psychiatry (b), Psychiatry (B), Hedi Chaker University hospital, sfax, Tunisia

**Keywords:** Depression, Elderly

## Abstract

**Introduction:**

Depression in the elderly represents a major public health problem, due to its high prevalence and its deleterious consequences in terms of morbidity and mortality, in particular by suicide.

**Objectives:**

1-Draw up the socio-demographic and clinical profile of elderly patients hospitalized in psychiatry for a major depressive episode 2-Determine the semiological and therapeutic characteristics of depression in the elderly.

**Methods:**

Participants were outpatients of Psychiatry B department in Hedi chaker University Hospital Center in Tunisia, over the age of 65, hospitalized in psychiatry for a major depressive episode, recruted between 2000 and 2015. The data was collected using a pre-established sheet containing socio-demographic information, the clinical and evolutionary characteristics of the depressive episode and the therapeutic data concerning the depressive episode.

**Results:**

30 patients were included in this study with an average age (69 Y) and sex ratio (0.66). More than half (53.3%, 16 patients) had a history of chronic somatic disease. The average length of hospitalization was 26 days. The most frequent reason for hospitalization is sadness of mood (43.3%) with cognitive impairment as the predominant clinical symptomatology (40%). 93.3% of the population received as treatment an antidepressant mainly Fluoxetine (50%).

**Conclusions:**

Depression and its different modes of expression in the elderly is a serious condition with direct effects on quality of life. Early detection is desirable in order to set up appropriate management, and thus prevent the occurrence of complications such as suicide.

